# Long non-coding RNA *HOTAIR* regulates cytoskeleton remodeling and lipid storage capacity during adipogenesis

**DOI:** 10.1038/s41598-022-14296-6

**Published:** 2022-06-16

**Authors:** Evdokiia Potolitsyna, Sarah Hazell Pickering, Thomas Germier, Philippe Collas, Nolwenn Briand

**Affiliations:** 1 Department of Molecular Medicine, Faculty of Medicine, Institute of Basic Medical Sciences, University of Oslo, Blindern, PO Box 1112, 0317 Oslo, Norway; 2 Department of Immunology and Transfusion Medicine, Oslo University Hospital, 0424 Oslo, Norway

**Keywords:** Actin, Long non-coding RNAs, Transcriptomics, Translation, Mesenchymal stem cells

## Abstract

The long non-coding RNA *HOTAIR* is the most differentially expressed gene between upper- and lower-body adipose tissue, yet its functional significance in adipogenesis is unclear. We report that *HOTAIR* expression is transiently induced during early adipogenic differentiation of gluteofemoral adipose progenitors and repressed in mature adipocytes. Upon adipogenic commitment, *HOTAIR* regulates protein synthesis pathways and cytoskeleton remodeling with a later impact on mature adipocyte lipid storage capacity. Our results support novel and important functions of *HOTAIR* in the physiological context of adipogenesis.

## Introduction

The distribution of adipose depots in the human body is of high significance for metabolic health^1^. The most differentially expressed gene between upper- and lower-body adipose tissue encodes the gluteofemoral-specific long non-coding RNA (lncRNA) *HOTAIR* (HOX Transcript Antisense RNA)^2,3^, within the *HOXC* locus. *HOTAIR* can be found in the nucleus, where it can bind chromatin and act as a scaffold for chromatin-modifying complexes^4^, and in the cytoplasm where it can promote ubiquitin-mediated proteolysis^5^ or function as a microRNA sponge^6^. The function of *HOTAIR* in adipose tissue, however, remains elusive. *HOTAIR* overexpression has been shown to promote adipogenesis in abdominal adipose stem cells (ASCs)^3^ and to prevent adipose differentiation of bone marrow mesenchymal stem cells^7^. In contrast to its effect in cancer cells^8^, *HOTAIR* overexpression does not affect ASC proliferation^3,7^, suggesting distinct mechanisms of action in adipose progenitors.

Besides the well-characterized adipogenic transcriptional cascade^9^, adipogenesis also entails major translational regulation^10^ and remodeling of the cytoskeleton^11^. Morphological changes from a fibroblast-like to a round, lipid-filled cell involve the disruption of F-actin stress fibers and the formation of a cortical actin network beneath the plasma membrane^12,13^. In mature adipocytes, lipid overloading induces a drastic remodeling of the actin cytoskeleton^14^ and affects the control of ribosomal protein expression downstream of insulin signaling^15^. Although recent evidence points to a functional link between translational regulation and cytoskeleton remodeling^16,17^, the molecular determinants coordinating these processes during early adipogenesis remain to be elucidated.

Here, we identify *HOTAIR* as a regulator of translation and of cytoskeleton reorganization during early adipogenesis in human ASCs, and provide evidence of a novel role of *HOTAIR* as a determinant of adipocyte lipid storage capacity.

## Results

### *HOTAIR* is upregulated during early adipogenesis

**Figure 1:**
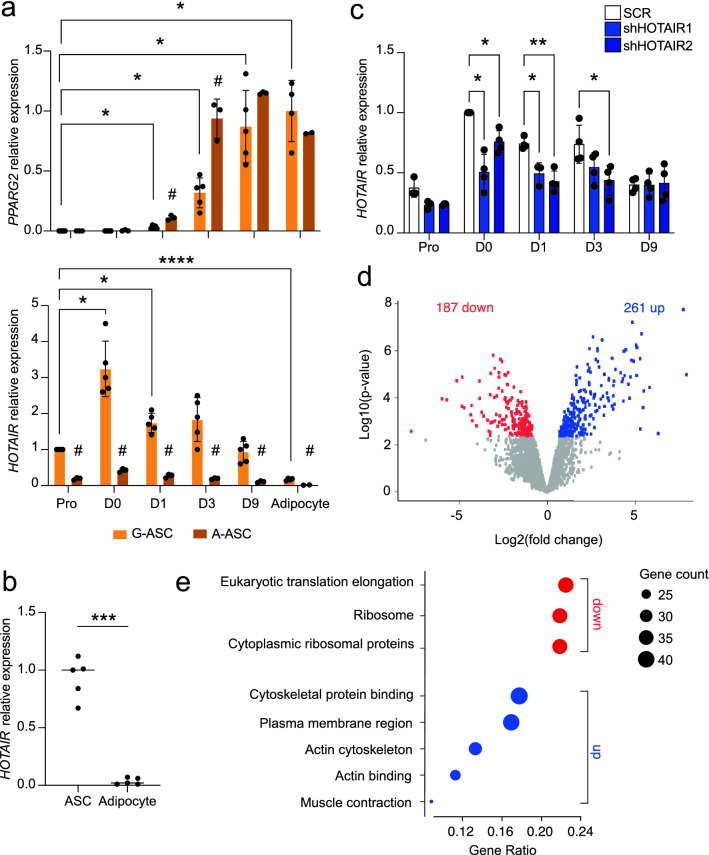
*HOTAIR* is upregulated during early adipogenesis. (**a**) Relative expression in G-ASCs and A-ASCs of *PPARG2,* normalized tomatched mature adipocytes, and of *HOTAIR*, normalized to proliferative G-ASCs (mean fold difference ± SD; *p < 0.05, **p < 0.005, ***p < 0.001, mixed effect analysis with Šídák's multiple comparisons test (G-ASC vs A-ASC) or Tukey's multiple comparisons test (time-course); ASCs n ≥ 3; adipocytes n ≥ 2). (**b**) Relative *HOTAIR* expression in proliferative ASCs vs. mature adipocytes from the same tissue (mean fold difference ± SD; ***p < 0.001, two-tailed paired t test; n = 5 adipose tissue depots from 3 independent donors). (**c**) RT-PCR analysis of *HOTAIR* expression in a control (SCR) and two stable *HOTAIR* KD G-ASC lines (shHOTAIR1 and 2) (mean fold difference ± SD; *p < 0.05, **p < 0.005, two-way ANOVA with Dunnett's multiple comparisons test; n ≥ 3). (**d**) Differential expression of the top 6000 highly expressed genes between SCR and two shHOTAIR G-ASC lines (adjusted *p*-values < 0.05, limma, n = 3). (**e**) Overrepresentation analysis of downregulated (red) and upregulated (blue) genes from (**d**) using GO, Wikipathways, Reactome and Hallmark gene sets from MSigDb (adjusted p-values < 1.2 × 10^−6^). The Gene Ratio is the fraction of differentially expressed genes in each gene set.

We first determined *HOTAIR* expression profile during adipogenic differentiation of gluteofemoral and abdominal ASCs (respectively G-ASCs and A-ASCs) from two donors, and in mature adipocytes isolated from the same tissues. ASCs from both donors show similar differentiation efficiency, based on induction of expression of the master adipogenic regulator *PPARG2* and of its targets (Fig. 1a; see supplementary Fig. S1a), and we confirm that *HOTAIR* expression is higher in G-ASCs compared to A-ASCs along the 9-day differentiation time course (Fig. 1a). Remarkably, *HOTAIR* expression is strongest in confluent G-ASCs (D0) and until differentiation day 3 (D3); however in contrast to previous findings, we find that adipogenesis elicits marked *HOTAIR* downregulation and accordingly, expression is lower in mature adipocytes than in ASCs isolated from the same tissue (Fig. 1a,b; see supplementary Fig. S1b).

LncRNAs are often involved in regulating neighboring protein-coding genes^18^; however, we show by RNA-seq that in differentiating G-ASCs, *HOTAIR* expression does not correlate with that of neighboring *HOXC* genes **(**see supplementary Fig. S2a, b**)**. To identify genes co-regulated with *HOTAIR*, we performed clustering of differentially expressed genes across time in G-ASCs (α < 0.01 between at least two consecutive time points) (see supplementary Fig. S2c). Enriched molecular function GO terms for the gene cluster containing *HOTAIR* relate to "extracellular matrix structural constituent” (GO:0005201) and “cytoskeletal binding protein” (GO:0008092) (see supplementary Fig. S2d).

To get insight into *HOTAIR* function, we stably silenced *HOTAIR* in G-ASCs using two small hairpin (sh)RNAs, both of which prevented *HOTAIR* upregulation at D0 and D1 (Fig. 1c, see supplementary Fig. S2e). Transcriptomic analysis of *HOTAIR*-silenced D0 G-ASCs reveals 187 significantly downregulated and 261 upregulated genes (Fig. 1d, see supplementary Fig. S2e and Table S5). Functional analysis shows that *HOTAIR* depletion results in the downregulation of translation-linked pathways and upregulation of actin cytoskeleton-related pathways, the amplitude of the effect being consistent with the knockdown efficiency (Fig. 1e, see supplementary Fig. S2f, Tables S6 and S7). Of note, genes upregulated in sh*HOTAIR* cell lines are not enriched for PRC2 or *HOTAIR* targets (see supplementary Fig.S2g, h and Table S7), indicating a PRC2-independent function of *HOTAIR* in G-ASCs. Together, these data suggest that *HOTAIR* is involved in the regulation of protein synthesis and actin cytoskeleton processes during early adipogenesis.

### *HOTAIR* knockdown alters translation and nucleoli morphology in proliferating G-ASCs

**Figure 2:**
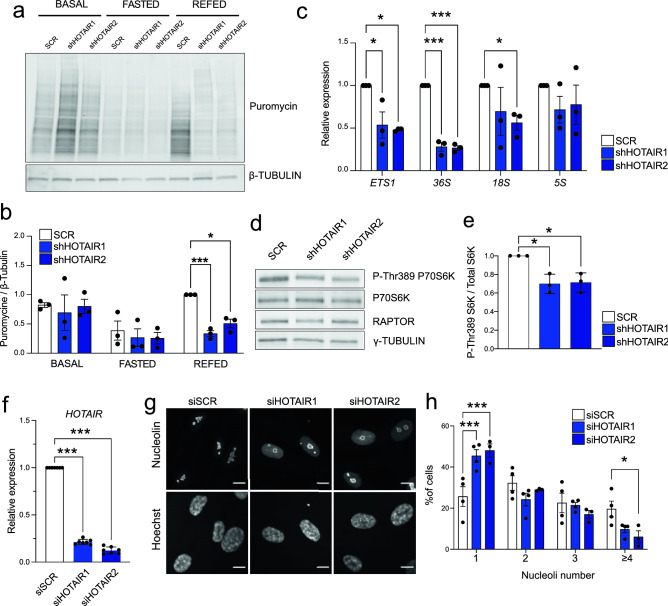
*HOTAIR* KD impairs refeeding-induced protein synthesis in proliferating G-ASCs. (**a**) SUnSET analysis of protein synthesis rates in basal, 24-h fasting (HBSS, 0.2% FFA-free BSA) and 24-h refeeding conditions in SCR, shHOTAIR1 and shHOTAIR2 G-ASCs (see Fig. S5 for original blots). (**b**) SUnSET quantification (*p < 0.05, ***p < 0.001, two-way ANOVA with Dunnett's multiple comparisons test;; n = 3). (**c**) RT-qPCR analysis of pre-rRNA (ETS1, 36S), 18S and 5S rRNA after 24-h refeeding (*p < 0.05, ***p < 0.001, two-way ANOVA with Dunnett's multiple comparisons test;; n = 3). (**d**) Western blot analysis of phospho-Thr389 P70S6K, total P70S6K, RAPTOR and γ-Tubulin protein levels after 24-h refeeding (see Fig. S6for original blots). (**e**) Quantification of phospho-Thr389 P70S6K/total P70S6K and RAPTOR/γ-Tubulin (*p < 0.05, two-tailed paired T test, n = 3). (**f**) RT-qPCR validation of transient *HOTAIR* KD in proliferating G-ASC (mean ± SD; *** *p* < 0.001, ANOVA with Holm-Sidak's multiple comparisons test; n = 7). (**g**) Immunostaining of Nucleolin in siSCR- and siHOTAIR1- and 2-transfected G-ASCs. Nuclei are stained with Hoechst. Scale bar: 10 µm. (**h**) Number of nucleoli per cell quantified from Nucleolin immunostaining in siSCR- and siHOTAIR1- and 2-transfected G-ASCs (mean ± SD; *p < 0.05, ***p < 0.001, two-way ANOVA with Dunnett's multiple comparisons test; n = 4 independent transfections; > 20 cells measured per experiment).

Because the translation machinery is strongly downregulated upon cell cycle arrest and during early adipogenesis^10^, we investigated the effect of *HOTAIR* KD on translation in proliferating G-ASCs. In basal culture conditions, sh*HOTAIR* G-ASCs present with normal cell morphology, and proliferation rates are unchanged (see supplementary Fig. S3a, b, c). Using a SUnSET assay, we find that while translation levels are unaffected in basal culture conditions, resumption of protein synthesis after a fasting and refeeding challenge is significantly impaired in sh*HOTAIR* G-ASCs (Fig. 2a, b). This coincides with a decrease in rDNA transcription in the refed state (Fig. 2c), a significant decrease in nucleolar volumes (see supplementary Fig. S3d–f), and a trend toward reduced nucleoli number (see supplementary Fig. S3g). In addition, phosphorylated P70S6K, a major regulator of protein synthesis in response to growth factors and nutritional cues^19^, is significantly decreased upon refeeding (Fig. 2d, e), consistent with the inability of sh*HOTAIR* G-ASCs to resume translation. Importantly, strong but transient downregulation of *HOTAIR* in G-ASCs using two distinct siRNAs (Fig. 2f) leads to a marked nucleoli remodeling, characterized by a morphological change and the redistribution of Nucleolin to the nucleolar periphery and into the nucleoplasm (Fig. 2g), and by a reduction of nucleoli number (Fig. 2h), altogether indicating nucleolar stress. We conclude that *HOTAIR* functions as a regulator of protein synthesis in G-ASCs.

### *HOTAIR* KD prevents cytoskeleton remodeling during adipogenesis

**Figure 3:**
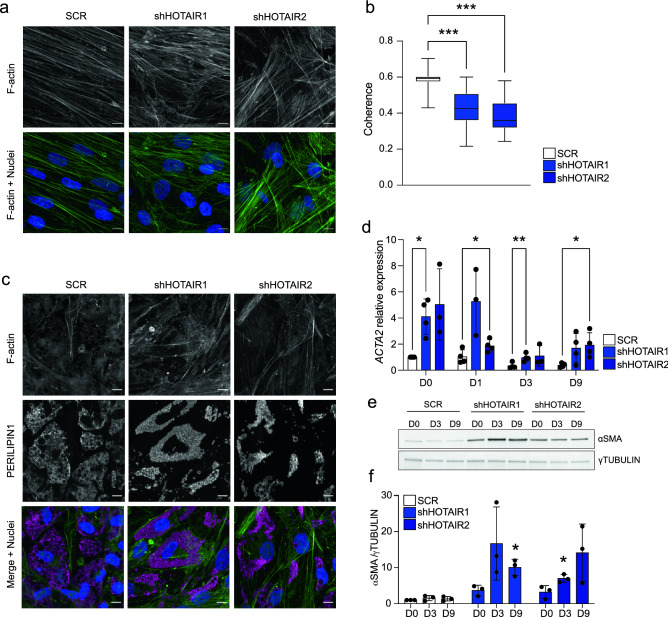
*HOTAIR* KD prevents cytoskeletal remodeling during adipogenesis.(** a**) Phalloidin staining of F-actin in D0 SCR and sh*HOTAIR* G-ASCs. Nuclei are stained with Hoechst. Scale bar: 10 µm. (**b**) Quantification of coherence in F-actin fibers alignment in D0 SCR and sh*HOTAIR* G-ASCs (***p < 0.0001, two-way ANOVA with Dunnett's multiple comparisons test; n = 3 experiments, with ≥ 4 fields measured per experiment, 5 measurements per field). (**c**) Phalloidin staining of F-actin (green) and immunostaining of PERILIPIN1 (magenta) in differentiated (D9) SCR and sh*HOTAIR* G-ASCs. Nuclei are stained with Hoechst. Scale bar: 10 µm. (**d**) Relative expression of *ACTA2* in differentiating scrambled control (SCR) and sh*HOTAIR* G-ASCs*,* normalized to SCR D0 (mean fold difference ± SD; *p < 0.05, **p < 0.005, two-way ANOVA with Dunnett's multiple comparisons test; n ≥ 3). (**e**) Western blot analysis (see Fig. S7for original blots) and (**f**) quantification of αSMA protein levels in differentiating SCR and sh*HOTAIR* G-ASCs (mean fold difference ± SD; *p < 0.05, two-way ANOVA with Tukey's multiple comparisons test; n = 3). γ-Tubulin is shown as a loading control.

Cytoskeleton remodeling is a key event in the morphological transition from ASCs to mature adipocytes^11,12,17^, and our transcriptomic analysis points to a deregulation of actin dynamics in ASCs knocked-down for *HOTAIR* (see Fig. 1e). In agreement, *HOTAIR* KD results in a disorganized F-actin network at D0, measured by a reduction in the orientation coherence of actin filaments (Fig. 3a, b). Upon differentiation (D9), we observe the expected reorganization of the F-actin network in control scrambled shRNA G-ASCs, while actin stress fibers persist in ASCs knocked-down for *HOTAIR* (Fig. 3c). This concurs with the overexpression of the α smooth muscle actin isoform (αSMA; encoded by *ACTA2*) in differentiating *HOTAIR* knocked-down ASCs, both at the mRNA and at protein levels (Fig. 3d–f). Thus, downregulation of *HOTAIR* expression during early adipogenesis impedes cytoskeleton remodeling, resulting in a persistent F-actin network at differentiation endpoint.

### *HOTAIR* KD results in impaired adipocyte lipid storage

**Figure 4:**
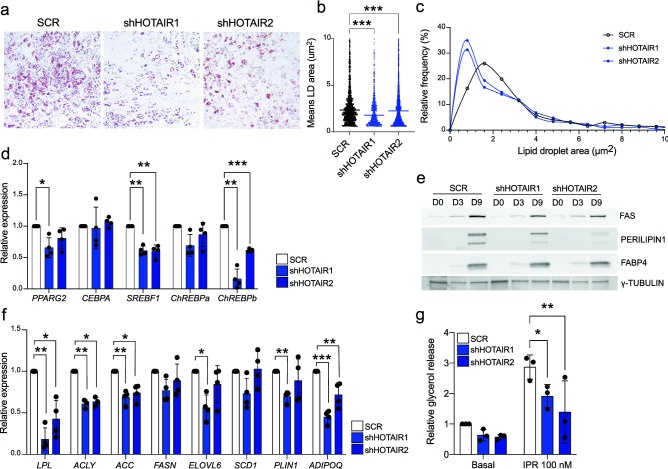
*HOTAIR* KD impairs lipid storage in mature adipocytes.(** a**) Oil red O staining (20X) of differentiated (D9) SCR and sh*HOTAIR* G-ASCs. LipidL droplet area analysis from PERILIPIN immunostainings of differentiated (D9) SCR and *HOTAIR* KD G-ASCs presented as (**b**) individual data scatter plot and mean (***p < 0.0001, two-way ANOVA with Dunnett's multiple comparisons test;; n = 3 experiments, with ≥ 3 fields measured per experiment) and (**c**) distribution frequency. (**d**) RT-qPCR analysis of adipogenic transcription factor gene expression at D9 (mean fold difference ± SD; *p < 0.05, **p < 0.005, ***p < 0.001, two-way ANOVA with Dunnett's multiple comparisons test; n = 4). (**e**) Western blot analysis of fatty acid synthase (FAS), PERILIPIN1, and FABP4 in differentiating SCR and shHOTAIR G-ASCs (representative blot from n = 3; see Fig. SS7for original blots). γ-Tubulin is shown as a loading control. (**f**) RT-qPCR analysis of glucose- and lipid uptake-related genes, de novo lipogenesis genes and mature adipocyte markers in differentiated (D9) SCR and shHOTAIR G-ASCs (mean fold difference ± SD; *p < 0.05, **p < 0.005, two-way ANOVA with Dunnett's multiple comparisons test; n = 4). (**g**) Fold induction of glycerol release in response to 100 nM isoproterenol stimulation for 2 h in differentiated (D9) SCR and shHOTAIR G-ASCs (mean fold difference ± SD; *p < 0.05, **p < 0.005, two-way ANOVA with Dunnett's multiple comparisons test; n = 3).

Actin cytoskeleton re-organization is required for both adipose differentiation and for the expansion of mature adipocyte lipid storage capacity^12–14^. In agreement, the defective cytoskeleton dynamics in shHOTAIR G-ASCs we observed above is associated with an overall decrease in lipid content at differentiation endpoint (Fig. 4a) and with significantly smaller lipid droplets (Fig. 4b, c). However, expression of the two master regulators of adipogenesis *PPARG2* and *CEBPA* (Fig. 4d), and their target FABP4 protein (Fig. 4e) is unaffected by *HOTAIR* KD. Thus, lipid accumulation or storage, rather than the adipogenic differentiation program per se, are defective after attenuation of *HOTAIR* expression. Accordingly, expression of *SREBF1* and *CHREBPb,* transcription factors mediating de novo lipogenesis gene induction, is significantly reduced by *HOTAIR* KD (Fig. 4d). Further, *HOTAIR* KD leads to reduced gene expression of key regulators of fatty acid availability (*LPL*), de novo fatty acid synthesis (*ACLY*, *ACC*), and triglyceride storage (*PLIN1*) (Fig. 4f, e), and to a blunted lipolytic response to isoproterenol, reflecting the global defect in lipid accumulation (Fig. 4g). Altogether, these results establish that *HOTAIR* functions as an early regulator of adipogenesis with determining effects on mature adipocyte lipid storage capacity.

## Discussion

We establish that *HOTAIR* expression is induced in the early phase of adipogenesis but strongly downregulated in mature adipocytes, and that this transient upregulation participates in the regulation of translation and of cytoskeletal remodeling during adipogenic commitment. Importantly, we find that deregulation of these processes later results in a reduced adipocyte lipid storage capacity.

LncRNA molecular function not only depends on their expression pattern and levels but is also determined by their subcellular localization^20^. LncRNA *HOTAIR* can bind DNA and act as an epigenetic scaffold in the nucleus, and interact with proteins and other RNAs in the cytoplasm. It is conceivable that siRNA-mediated *HOTAIR* silencing differentially affects distinct pools of *HOTAIR,* due to variation in their location, binding partners, or stability^21^. Surprisingly, the transcripts we find deregulated upon *HOTAIR* silencing are not enriched for PRC2 target genes, a well-described *HOTAIR* partner in the nucleus^22^. Instead, we describe a defective cytoskeletal remodeling during early adipogenesis, in line with the recently reported deregulation of the Rho/ROCK-mediated remodeling of actin cytoskeleton in abdominal ASCs upon *HOTAIR* overexpression^23^. Such modulation of Rho GTPases signaling, plausibly through *HOTAIR* cytoplasmic miRNA-sponge function^24^, could account for the downstream transcriptomic defects observed upon HOTAIR silencing.

*HOTAIR* knockdown in proliferating ASCs affects nucleolus morphology and results in an inability to resume translation upon nutrient availability, which correlates with a decrease in mTORC1 signaling. Regulation of mTOR signaling by *HOTAIR* has been reported^25,26^, although the mechanistic basis for this regulation remains unknown. Interestingly, mTORC1 activation by nutrients is supported by its association with focal adhesions and with the actin cytoskeleton^27^, suggesting that cytoskeletal alterations might underlie the mTORC1 signaling defect we detect after attenuation of *HOTAIR* expression in ASCs.

An active cytoskeleton remodeling during adipogenesis is necessary to regulate adipocyte formation and accommodate lipid droplet expansion^12–14^. We find that preventing *HOTAIR* transient upregulation upon adipogenic commitment results in a defective actin cytoskeleton reorganization. This phenotype persists at later differentiation stages and is accompanied by a drastic reduction of lipid droplet size, and altered de novo lipogenesis downstream of SREBP1 and ChREBPβ. While *SREBF1* downregulation could result from compromised mTOR signaling^19,28^, altered dynamics of the actin cytoskeleton and increased mechanical constraints could also directly account for a reduction of SREBP1 activity^29^.

Adipose tissue depots differ by their mechanisms of expansion, with lower-body adipose tissue having the capacity to rapidly increase in mass through ASC differentiation, rather than adipocyte hypertrophy^30^. ASCs commitment towards the adipogenic lineage is controlled by both the niche and intrinsic cellular mechanical properties^31^. Therefore, depot-specific remodeling mechanisms might be linked to distinct macro-architecture^32^ and cellular structure^33,34^. In gluteofemoral ASCs, *HOTAIR*-mediated regulation of the cytoskeletal tensional state might preserve ASC differentiation capacity, thereby promoting healthy lower-body adipose tissue expansion.

*HOTAIR* downregulation in ASCs results in the downregulation of translation-related genes, and in the upregulation of cytoskeletal genes, a phenotype that strikingly mirrors that of *Zc3h10* silenced preadipocytes^17^. In both models, deregulation of these coordinated cellular processes upon adipogenic commitment leads to an altered lipid storage capacity in differentiated adipocytes. Deregulation of the translation machinery and of the cytoskeleton occurs in both lipoatrophy^12,35^ and obesity^14,15^, highlighting the importance of these processes in the pathophysiology of adipose tissue.

## Methods

### Primary cells isolation

This study was approved by and conducted in agreement with the guidelines and regulations of the Regional Committee for Research Ethics for Southern Norway. ASCs were isolated from liposuction material from subcutaneous gluteal or abdominal adipose tissue from five healthy women (age 28–50; BMI 22–27 kg/m^2^) after informed consent was given. After washing in Hank’s balanced salt solution (HBSS), tissues were dissociated for 50 min at 37 °C with 1% collagenase (Worthington #LS004204). Adipocytes were separated from stromal vascular (SV) cells after sedimentation at 300 × *g* for 10 min and lysed in TRIzol (Invitrogen) before RNA isolation. ASCs were further selected by differential plating.

### Cell culture and differentiation

ASCs were cultured in DMEM/F12 (17.5 mM glucose) with 10% fetal calf serum and 20 ng/ml basic fibroblast growth factor (Pro). Upon confluency, growth factor was removed and cells were cultured for 72 h before induction of differentiation (D0). For adipose differentiation, ASCs were induced with a cocktail of 0.5 µM 1-methyl-3 isobutyl xanthine, 1 µM dexamethasone, 10 µg/ ml insulin and 200 µM indomethacin. Differentiation media was renewed every 3 days, and samples harvested 1, 3, and 9 days after induction. Cells were stained with Oil Red-O on D9. All differentiation experiments were done in at least three biological replicates between passage 4 and 9.

### HOTAIR knockdown

For transient *HOTAIR* knockdown (KD), gluteofemoral ASCs were electroporated with 30 nM of one of the two siRNAs (siHOTAIR 1: N272221 or siHOTAIR 2: N272230; Ambion). Lonza Nucleofector device was used with a human MSC nucleofection kit (VVPE-1001; Lonza) according to manufacturer instructions. After electroporation, cells were cultured for 72 h before harvesting RNA. Stable *HOTAIR* KD was achieved by expression of two shRNAs (Table S1) cloned into a pLV-hU6-EF1a-bsd vector (Biosettia). Second generation lentiviral packaging system (plasmids psPAX2 #12,260 and pCMV-VSV-G #8454; Addgene) was used to produce virus in LentiX cells (632,180; Takara Bio). Media containing viral particles was filtered with a 0.45 µm filter and equal volumes of this media were used to infect ASC together with 8 µg/ml Polybrene (TR-1003-G, Merck). Infected cells were selected for 7 days in 10 µg/ml Blasticidin (ant-bl-05, Invivogen).

### Gene expression

RNA was isolated using RNeasy kit (QIAGEN) and 1 µg was used for cDNA synthesis using High-Capacity cDNA Reverse Transcription Kit (ThermoFisher). RT-PCR was done using IQ SYBR green (Bio-Rad Laboratories) with *SF3A1* as a reference gene. PCR conditions were 95 °C for 3 min and 40 cycles of 95 °C for 30 s, 60 °C for 30 s, and 72 °C for 20 s followed by a melting curve. PCR primers are listed in Table S2.

### Protein expression

Proteins were resolved by gradient 4–20% SDS–PAGE, transferred onto Immobilon-FL (Millipore) or nitrocellulosis 0.45 μm (Biorad) membranes and blocked with Odyssey blocking buffer (LI-COR). Membranes were cut horizontally to allow for multiple parallel hybridizations. Membranes were incubated with antibodies overnight at 4 °C (Table S3). Proteins were visualized using IRDye-800-, IRDye-680-, or HRP-coupled secondary antibodies. Bands were quantified by densitometry (Image Lab; BioRad).

### RNA sequencing analysis

Paired-end Illumina RNA sequencing (RNA-seq) was done in biological triplicates for 2 independent donors. Reads were filtered with fastp, aligned to the hg38 genome (ensembl v95 annotation) with hisat2, counted using featureCounts (–fraction -M), and differential gene expression was analyzed using limma^36^ (Table S4). Genes differentially expressed between SCR and both shHOTAIR G-ASC lines with a FDR adjusted *p*-value < 0.05 are shown in Table S5. Differentially expressed genes with high average expression (within the top 6000 genes) were tested for overrepresentation against GO, KEGG, Wikipathways, Reactome and Hallmark gene sets from MSigDb and terms with a FDR adjusted *p*-value < 0.01 are presented in Tables S6 and S7. DPGP^37^ was used to cluster genes with differential expression (FDR adjusted *p*-value < 0.01) across adipogenesis in native G-ASCs (Fig. S2c).

### Lipolysis and SUnSET assays

To assess lipolysis, G-ASCs on D9 were serum-deprived overnight. Glycerol release in response to 100 nM isoproterenol was measured in the medium (GY105; Randox) and normalized to well protein content. SUnSET assay was done by labeling newly synthesized proteins with 1 µM Puromycin for 1 h followed by Western blotting using anti-Puromycin antibodies (Table S3).

### Imaging

For immunofluorescence, cells were fixed in 4% paraformaldehyde, permeabilized in PBS/0.1% Triton X-100/0.01% Tween 20/2% BSA, incubated with relevant antibodies and stains (Table S3) and mounted (DAKO S3023, Agilent). Fluorescence images were taken on a Dragonfly microscope (Oxford Instruments; 60 × 1.4 NA objective; 4-μm stack with 0.3-μm steps).

### Image analysis

Nucleolar volumes and numbers were measured using Nemo software with the nucleolin channel used as a nucleolar marker. The number of nucleoli was corrected manually in the transient KD analysis due to major redistribution of nucleolin to the nucleoplasm preventing correct segmentation of nucleoli in some cases. Morphological Segmentation plugin from MorphoLibJ collection of plugins in ImageJ was used to define lipid droplets from perilipin-1 immunostaining, “border object” and connectivity of 8 settings were used (https://imagej.net/plugins/morpholibj). Lipid droplet area was then measured with the “Analyse particles” function with a threshold on circularity and size. Actin cytoskeleton coherence was measured from phalloidin staining with the OrientationJ plugin (http://bigwww.epfl.ch/demo/orientation/). Cell size and confluency were measured from phase-contrast images with Morphological Segmentation and PHANTAST plugins. Raw unmodified images were used for all the analyses except for phase-contrast images which were adjusted for contrast and Gaussian blur was used to enhance the segmentation quality.

## Data availability

RNA-seq data are available from Gene Expression Omnibus (GEO) accession GSE176020.

## Supplementary Information

Supplementary Information 1.

Supplementary Information 2.
