# Supplementary material for: Long non-coding RNA HOTAIR regulates cytoskeleton remodeling and lipid storage capacity during adipogenesis
Source: Sci Rep. 2022 Jun 16;12:10157. doi: 10.1038/s41598-022-14296-6 (PMC9203762; doi:10.1038/s41598-022-14296-6)
Supplement: Supplementary file 1 — Supplementary Information 1. [file 41598_2022_14296_MOESM1_ESM.pdf]

**Supplementary information**

**Supplemental Tables and Figures**

| shRNA | Sequence |
|---|---|
| **shHOTAIR1** | AAAAGGGAGTACAGAGAGAATAATTGGATCCAATTATTCTCTCTGTACTCCC |
| **shHOTAIR2** | AAAAGCGAACCACGCAGAGAAATTTGGATCCAAATTTCTCTGCGTGGTTCGC |

**Table S1: *HOTAIR* shRNA sequences**

| Gene | Forward primer | Reverse primer |
|---|---|---|
| ***HOTAIR*** | GGAGCCCAGAGTTACAGACG | TCAGACTCTTTGGGGCCTTA |
| ***PPARG2*** | AAGCGATTCCTTCACTGATACAC | CTTCCATTACGGAGAGATCCAC |
| ***CEBPA*** | CCCAGAGGGACCGGAGTTAT | AGACGCGCACATTCACATTG |
| ***SREBF1*** | GGAGCCATGGATTGCACTTT | GTCAAATAGGCCAGGGAAGTCA |
| ***ChREBPa*** | AGTGCTTGAGCCTGGCCTAC | TTGTTCAGGCGGATCTTGTC |
| ***ChREBPb*** | AGCGGATTCCAGGTGAGG | TTGTTCAGGCGGATCTTGTC |
| ***LPL*** | CCGCCGACCAAAGAAGAGAT | TAGCCACGGACTCTGCTACT |
| ***ACLY*** | TCTTTGTCCGAAGAGGTGGC | GCCAAAGACATGGATGGGGA |
| ***ACC*** | CTGCAAAATGAAGGGGAGCG | GGTTACAGTCAGTGCGGACA |
| ***FASN*** | CTTCAAGGAGCAAGGCGTGA | ACTGGTACAACGAGCGGATG |
| ***ELOVL6*** | TCAGCAAAGCACCCGAAC | AGCGACCATGTCTTTGTAGGAG |
| ***SCD1*** | ACACCCAGCTGTCAAAGAGA | GCCAGGTTTGTAGTACCTCCTC |
| ***PLIN1*** | GACAAGGAAGAGTCAGCCCC | GAGAGGGTGTTGGTCAGAGC |
| ***ADIPOQ*** | TGTGGTTCTGATTCCATACCAG | CGGGCAGAGCTAATAGCAGTA |
| ***SF3A1*** | AGGGTCCAGTGTCCATCAAA | AGAGACCTGGTCCGTGAGTG |

**Table S2: RT-qPCR primers**

| | Reference | Western Blot | IF |
|---|---|---|---|
| **Nucleolin** | Abcam, ab22758 | | 1/1000 |
| **Perilipin1** | Progen, GP29 | 1/1000 | 1/500 |
| **FABP4** | Santa Cruz sc-271529 | 1/1000 | |
| **γ-Tubulin** | Sigma T5326 | 1/10000 | |
| **FAS** | Santa Cruz, sc-8009 | 1/1000 | |
| **P-Thr389P70S6K** | Cell signaling, #9234 | 1/1000 | |
| **P70S6K** | Cell signaling, #9202 | 1/1000 | |
| **RAPTOR** | Cell signaling, #2280 | 1/1000 | |
| **Puromycin** | Millipore, MABE343 | 1/10000 | |
| **β-Tubulin** | Abcam, #6046 | 1/1000 | |
| **Phalloidin-iFluor 594 Reagent** | Abcam, #ab176757 | | 1/1000 |
| **Phalloidin-iFluor 647 Reagent** | Abcam, #ab176759 | | 1/1000 |
| **Hoechst 33342** | ThermoFisher, 62249 | | 2 ug/ml |

**Table S3: Antibodies and stains**

| Tool | URL | Reference |
|---|---|---|
| **Hisat2** | http://daehwankimlab.github.io/hisat2/ | 1 |
| **FeatureCounts** | http://subread.sourceforge.net/ | 2 |
| **EdgeR** | https://bioconductor.org/packages/release/bioc/html/edgeR.html | 3 |
| **Limma** | https://bioconductor.org/packages/release/bioc/html/limma.html | 4 |

**Table S4: Bioinformatics packages**

**Table S5: List of differentially expressed genes between SCR et both shHOTAIR G-ASC lines**
Genes with a FDR adjusted p-value < 0.05 are shown.

**Table S6: Overrepresentation analysis of genes upregulated in shHOTAIR cell lines**
Upregulated genes with high average expression (within the top 6000 genes) were tested for overrepresentation against GO, KEGG, Wikipathways, Reactome and Hallmark gene sets from MsigDb. Terms with a FDR adjusted p-value < 0.01 are presented.

**Table S7: Overrepresentation analysis of genes downregulated in shHOTAIR cell lines**
Downregulated genes with high average expression (within the top 6000 genes) were tested for overrepresentation against GO, KEGG, Wikipathways, Reactome and Hallmark gene sets from MsigDb. Terms with a FDR adjusted p-value < 0.01 are presented.

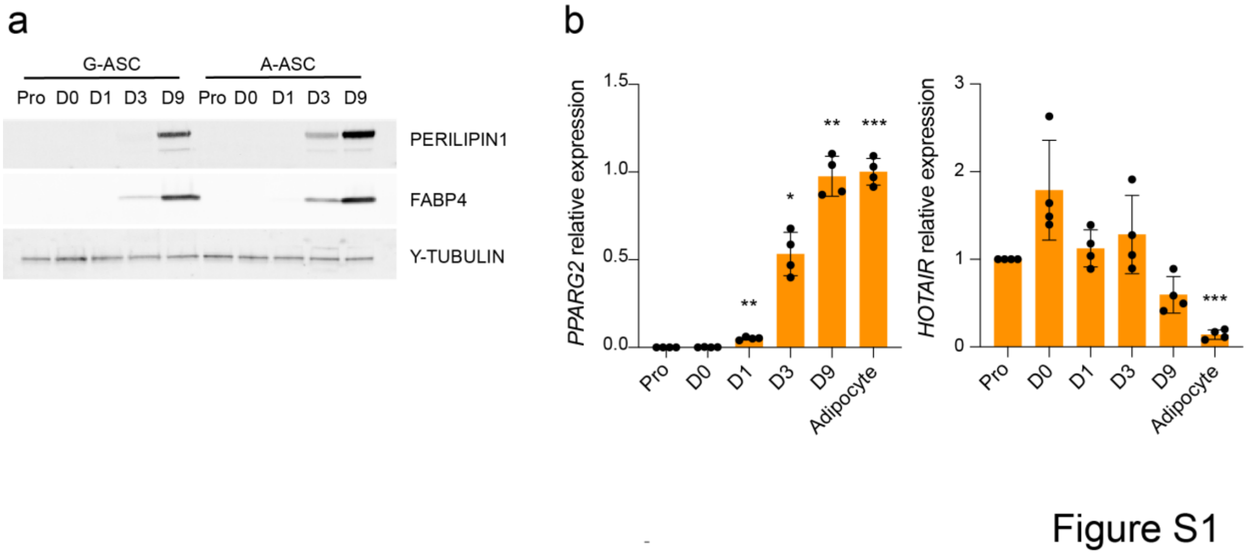


***HOTAIR* expression is transiently induced during adipogenesis**. **a** Western Blot analysis of Perilipin 1 and FABP4 protein expression in differentiating G-ASCs and A-ASCs**.** γ-Tubulin is shown as a loading control**. b** Relative expression of *PPARG2* normalized to paired mature adipocytes and *HOTAIR* normalized to proliferative G-ASCs from a second independent donor (Donor 2; mean fold difference ± SD; *p < 0.05, **p < 0.005, ***p < 0.001, two-way ANOVA, n = 4).

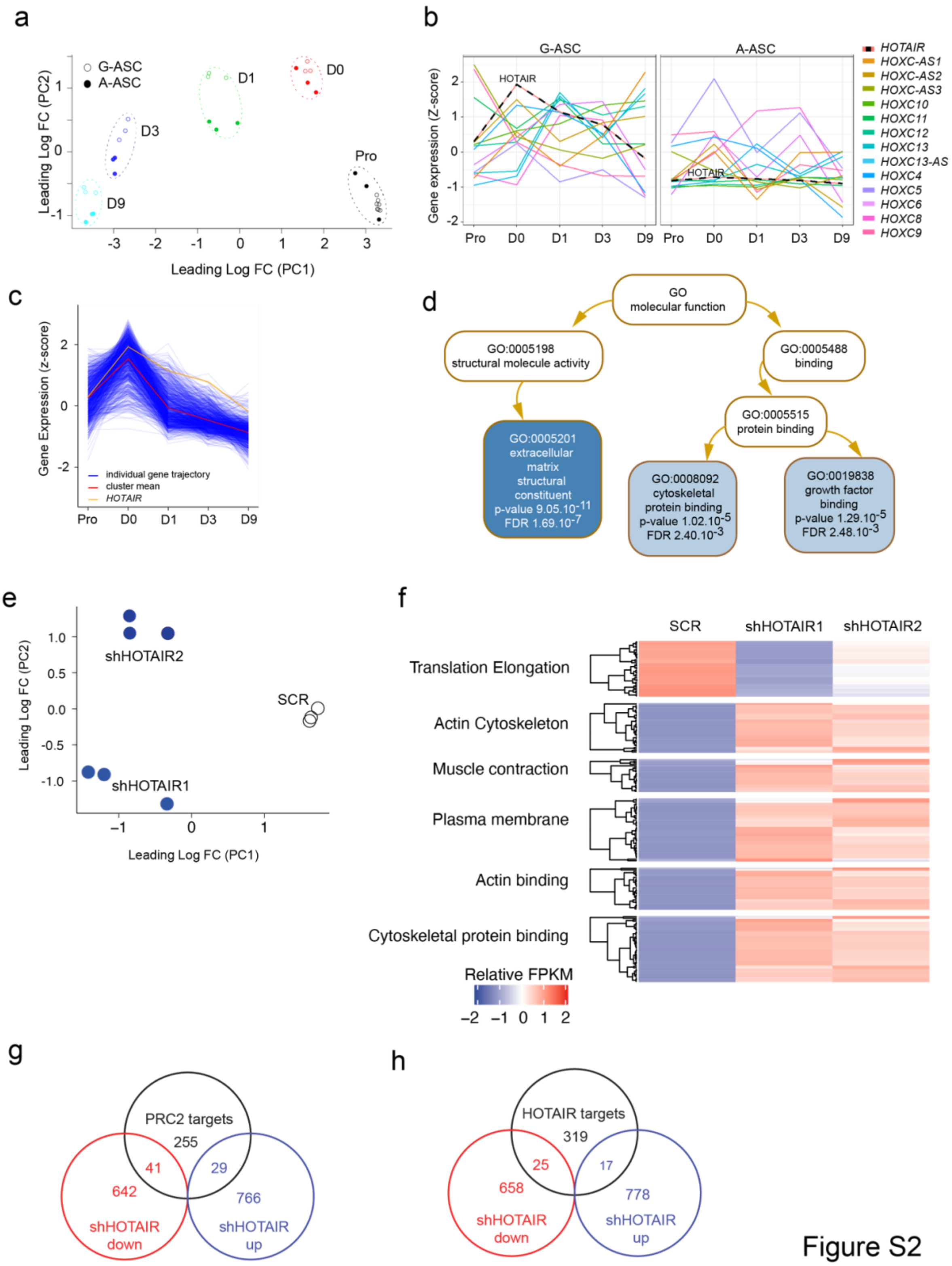


Figure S2

***HOTAIR* coexpression analysis during adipogenesis a** Principal component analysis of gene expression during adipose differentiation of G-ASCs and A-ASCs. **b** Expression profile of *HOXC* cluster genes in G-ASCs and A-ASCs. **c** Expression profile of all differentially expressed genes in the *HOTAIR*-containing cluster. **d** Enriched molecular function GO terms for the gene expression cluster shown in (**c**). **e** Principal component analysis of gene expression at D0 during in control (SCR) and two stable *HOTAIR* KD G-ASC lines (shHOTAIR1 and 2). **f** Heatmap of genes for the top enriched GO terms (see Fig.1e) in SCR, shHOTAIR1 and shHOTAIR2 G-ASC. Venn diagrams showing the proportion of PRC2 target genes5 (**g**) and *HOTAIR* target genes6 (**h**) deregulated in shHOTAIR G-ASCs.

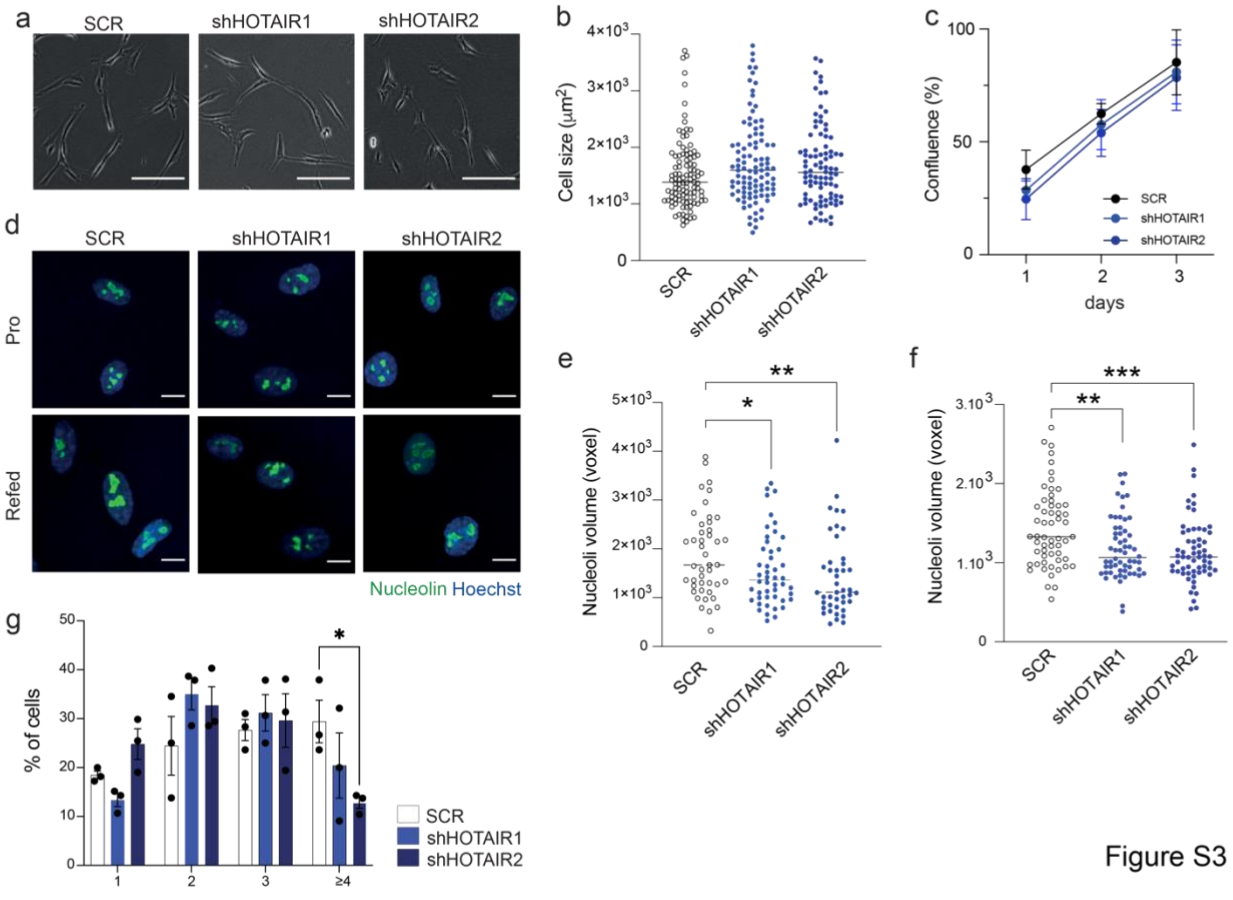


**Cell and nucleolar morphology of G-ASCs after stable *HOTAIR* KD**. **a** Phase contrast images and **b** cell size measurements of control (SCR) and two stable *HOTAIR* KD G-ASC lines (shHOTAIR1 and 2) (two-way ANOVA with Dunnett's multiple comparisons test, n = 2; non-significant differences). Scale bar: 100 µm. **c** Cell confluence over time measured on phase contrast images of SCR, shHOTAIR1 and 2 G-ASCs in basal proliferating state (Pro) (two-way ANOVA with Dunnett's multiple comparisons test, n = 4; non-significant differences). **d** Immunostaining of nucleolin in SCR and shHOTAIR1 and 2 G-ASCs in basal proliferating state (Pro) and after fasting and refeeding (Refed). Nuclei are stained with Hoechst. Scale bar: 10 µm. **e** Nucleolar volume quantification in SCR, shHOTAIR1 and shHOTAIR2 G-ASCs in basal conditions, and **f** after fasting and refeeding (*p < 0.05, **p < 0.01, ***p < 0.001, two-way ANOVA with Tuckey's multiple comparisons test, n = 3). **g** Number of nucleoli per cell quantified from Nucleolin immunostaining in SCR and shHOTAIR1 and 2 G-ASCs (mean ± SD; *p < 0.05, two-way ANOVA with Dunnett's multiple comparisons test; n = 3).

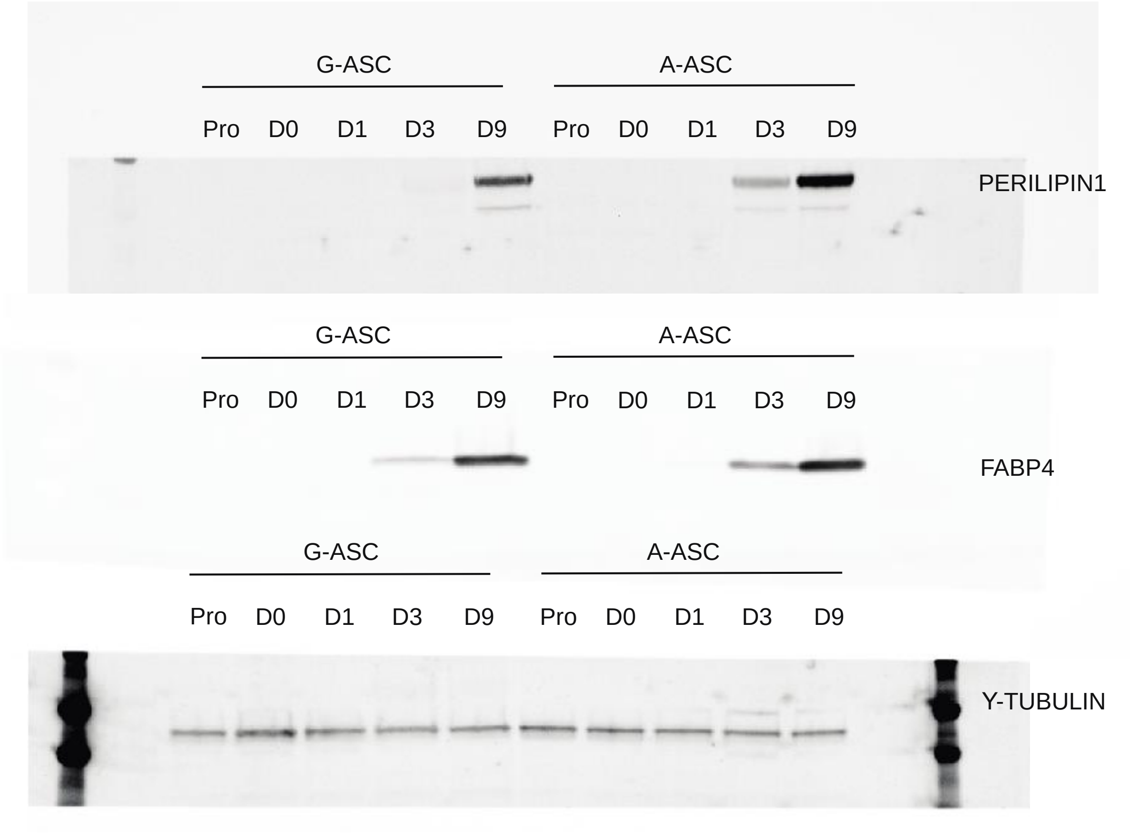


**Figure S4**: Uncropped blots for Fig.S1a

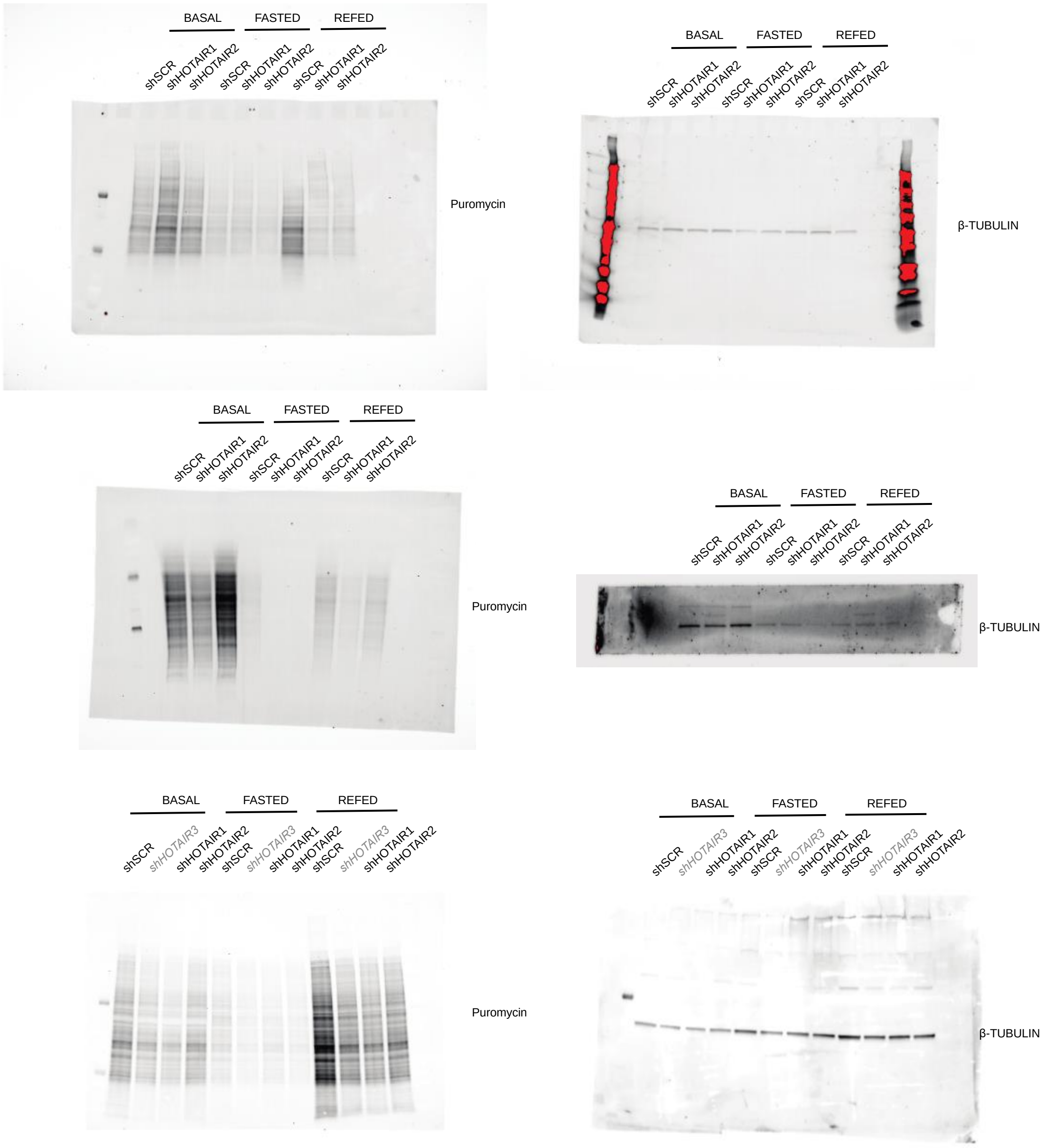


**Figure S5**: Uncropped blots for Fig.2a

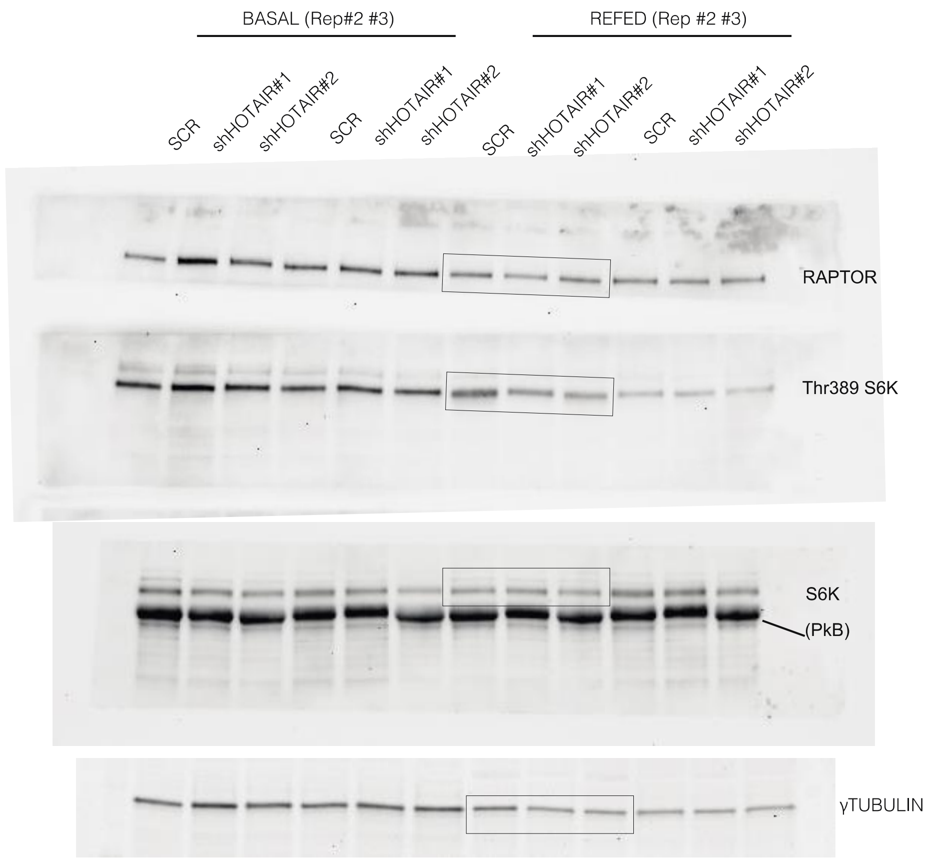


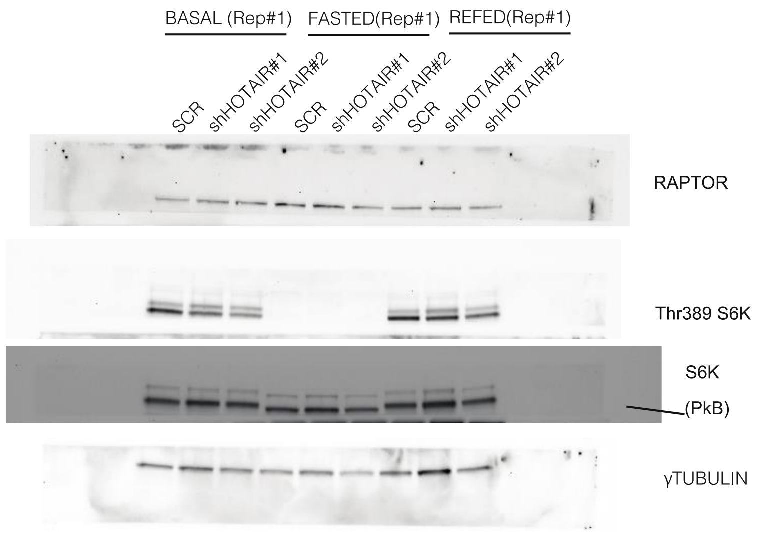


**Figure S6**: Uncropped blots for Fig.2d

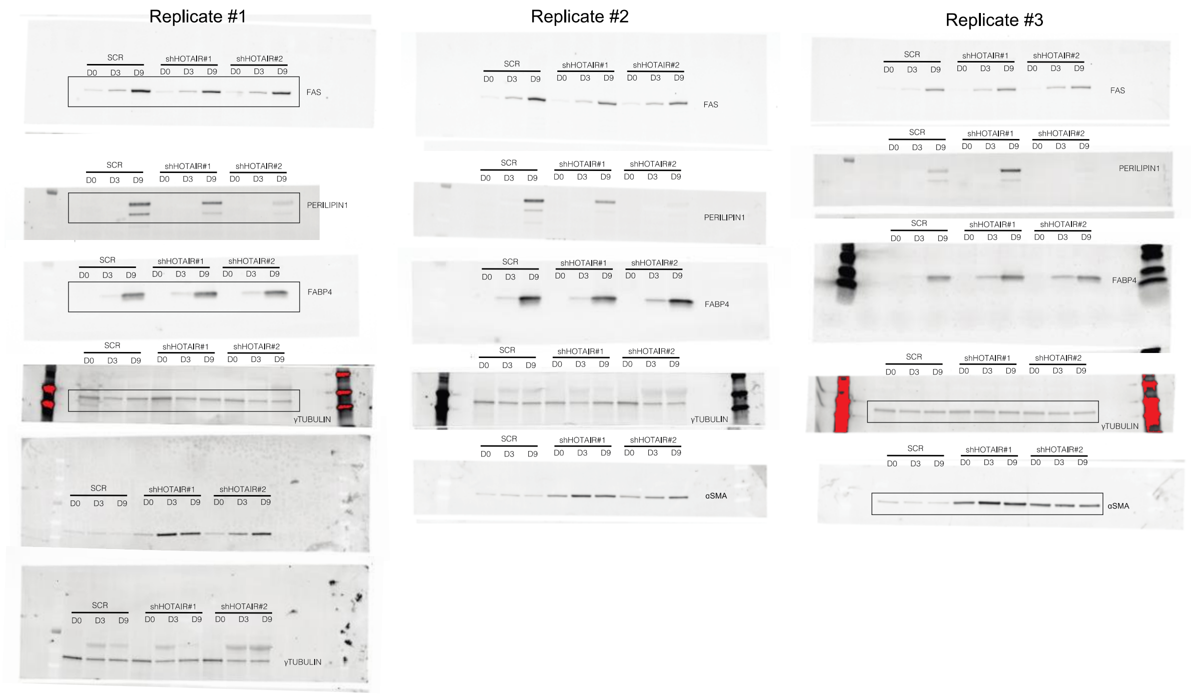


**Figure S7**: Uncropped blots for Fig.3e and 4d

**Supplemental References**


1. Kim, D., Paggi, J. M., Park, C., Bennett, C. & Salzberg, S. L. Graph-based genome alignment and genotyping with HISAT2 and HISAT-genotype. *Nat. Biotechnol.* **37**, 907–915 (2019).
2. Liao, Y., Smyth, G. K. & Shi, W. featureCounts: an efficient general purpose program for assigning sequence reads to genomic features. *Bioinformatics* **30**, 923–930 (2014).
3. Robinson, M. D., McCarthy, D. J. & Smyth, G. K. edgeR: a Bioconductor package for differential expression analysis of digital gene expression data. *Bioinformatics* **26**, 139–140 (2010).
4. Ritchie, M. E. *et al.* limma powers differential expression analyses for RNA-sequencing and microarray studies. *Nucleic Acids Res.* **43**, e47 (2015).
5. Ben-Porath, I. *et al.* An embryonic stem cell-like gene expression signature in poorly differentiated aggressive human tumors. *Nat. Genet.* **40**, 499–507 (2008).
6. Chu, C., Qu, K., Zhong, F. L., Artandi, S. E. & Chang, H. Y. Genomic maps of long noncoding RNA occupancy reveal principles of RNA-chromatin interactions. *Mol. Cell* **44**, 667–678 (2011).
